# Methods for a Survey of Overweight and Obesity Coordinated With Oral Health Surveillance Among Ohio Third-Grade Students

**Published:** 2008-12-15

**Authors:** Elizabeth J. Conrey, Erinn M. Hade, Angela Norton, Heidi Scarpitti

**Affiliations:** Ohio Department of Health, State Epidemiology Office; The Ohio State University, Columbus, Ohio; Ohio Department of Health, Columbus, Ohio; Ohio Department of Health, Columbus, Ohio

## Abstract

**Introduction:**

Data on overweight and obesity prevalence among children enable state and local officials to develop, target, fund, and evaluate policies and programs to address childhood overweight. During the 2004-2005 school year, the Ohio Department of Health (ODH) conducted surveillance of elementary school-aged children through coordination with the ODH oral health survey to create a system that would provide county and state estimates of obesity and overweight prevalence.

**Methods:**

We used a stratified, cluster-sampling survey design. Schools were considered clusters and were sampled from strata determined by their county and by their participation rate in the Free and Reduced Price Meal program. We selected public elementary schools by probability proportional to size sampling without replacement. We requested consent from the guardian or parent of each third-grade student. Trained health care professionals used state-purchased equipment to weigh students and measure their height. We removed implausible observations and calculated sex-specific, body mass index (BMI)-for-age percentiles using Centers for Disease Control and Prevention growth charts.

**Results:**

Of eligible schools, 374 agreed to height and weight screening; 41 were considered substitutes. Of 26,590 enrolled students, 17,557 (66.0%) returned consent forms, and 15,209 (57.2%) provided consent. BMI estimates were generated for 14,451 students, resulting in an overall response rate of 54.3%. The overall oral health response rate was 52.8%.

**Conclusion:**

By adding BMI screening to Ohio's third-grade oral health survey and incorporating trained volunteer screeners, the ODH successfully implemented overweight and obesity surveillance using minimal resources. Future efforts should focus on improving student response rate.

## Introduction

Obesity among children aged 6 to 11 years in the United States more than tripled from 1980 through 2002 (1,2). Although there is evidence that the rising trend has subsided, prevalence remains high; from 2003 through 2006, 17% of children aged 6 to 11 were obese (3). Overweight and obesity are public health concerns because they affect children's current and future health. Obese children (body mass index [BMI] ≥95th percentile for age) are more likely than children of a healthy weight (BMI 5th percentile to <85th percentile) to have high blood pressure and high cholesterol, lipid, and fasting insulin levels (4,5), even though medical complications may not become clinically apparent for decades (6). Furthermore, obese children are likely to become obese adults (7-11). After age 3, being overweight or obese increases the probability of being obese as a young adult. The probability increases with the age of the child and is higher at all ages for obese children than for overweight children (9).

Data on overweight and obesity prevalence among children enable state and local officials to develop, target, fund, and evaluate policies and programs to address childhood overweight. Some states may have access to overweight and obesity data for low-income children aged less than 5 years through the Centers for Disease Control and Prevention's (CDC's) Pediatric Nutrition Surveillance System (PedNSS) and to self-reported data for children in grades 9 through 12 through the Youth Risk Behavior Surveillance System (YRBSS). However, data for overweight and obesity prevalence among elementary school-aged children are sparse. A growing number of states have begun to collect and report this information (12-16).

By the spring of 2004, community members in Ohio had a growing interest in childhood obesity, and some schools had begun measuring students' heights and weights in an effort to collect data on BMI. The Ohio Department of Health (ODH) began to explore ways to provide local-level data on childhood obesity. ODH was also dedicated to producing accurate data and, therefore, took measures to guarantee uniform use of appropriate equipment, techniques, and BMI calculation and to ensure confidentiality and appropriate communication to families. However, the state had a limited amount of money and personnel to contribute to this effort.

One opportunity explored for collecting and providing the data was to collaborate with the ODH Bureau of Oral Health Services during the administration of its oral health survey (17). The oral health survey provides state-level and county-level data on the oral health status of third-grade students approximately every 5 years (most recently for the 1999-2000 and 2004-2005 school years) and annual data at the state level. Data collection consists of obtaining information from parents and conducting a physical screening of students at school. After making a commitment to the Bureau of Oral Health Services not to disrupt the quality of the oral health data collection process, ODH expanded the oral health survey during the 2004-2005 school year to include height and weight screening. ODH's School and Adolescent Health Services added anthropometric data to the oral health data collection process to provide county-level and regional-level estimates of obesity and overweight prevalence among elementary school-aged children and to identify characteristics associated with childhood overweight and obesity in Ohio.

## Methods

### Sampling design

Our sampling frame was all 1,994 public schools in the state of Ohio that were not community schools (community schools are independently operated, publicly funded, tuition-free public schools that are created on the basis of a contract or “charter”). We used a stratified, cluster-sampling survey design, in which schools were considered clusters. We divided schools into 2 groups: 1) by county, and 2) by free and reduced-price meal (FRPM) program participation rate. We chose schools within each stratum by probability proportional to size sampling. We also selected potential replacement schools at this time. For ease of implementation, we screened all third-grade classrooms in each selected school.

Stratification variables were chosen 1) to be sure that each county was represented in the sampling and to provide estimates for each county, and 2) because participation in the FRPM program is a proven indicator of oral health and because participation varies widely among counties (18). We divided 62 of the 88 Ohio counties into 2 strata: 1) schools with less than 50% of students participating in the FRPM program and 2) schools with 50% or more students participating in the FRPM program. At the time this survey was designed, 30 schools from various counties did not have available information about the percentage of students enrolled in the FRPM program; we chose a small subset of these schools to survey.

The oral health sampling was designed for precision of dental health variables; sample size estimates for the number of schools to sample within each county were calculated from dental caries data from the 1998-1999 survey. The number of schools sampled for each county was calculated to be almost certain (α = .001) of estimating the total number of Ohio third-grade students with dental caries to within 10% of the true value in a 1-stage cluster sample, given previous estimates (19). Height and weight measurements were added to the oral health survey after its sampling methodology was finalized for 2004-2005.

### Study population and schools

We sent district superintendents for selected schools a letter signed by the directors of the Ohio Department of Education (ODE) and the ODH to obtain approval for school district participation. One of 11 ODH oral health screeners then scheduled onsite meetings with principals of selected schools. Principals were briefed on the study and told that neither individual-level nor school-level BMI data would be shared with faculty members or with families. If the principal consented to participation, the screening was scheduled for a date within the 2004-2005 school year (ie, September of 2004 through June of 2005). Each principal received a nutrition and physical activity educational packet for distribution to all classrooms. If a school refused to participate, we contacted replacement schools until a school was found that agreed to participate. (Replacement schools were also needed if superintendent consent was not obtained, when schools closed, or when third-grade classes were removed from the schools originally sampled.)

Classroom teachers were instructed to send home ODH-provided consent forms with all third-grade students approximately 2 to 3 weeks before the screening date. A signed consent form from a parent or guardian was required for each student to participate. Some schools sent a second letter home to encourage participation. On each consent form, a parent or guardian reported the student's age, birth date, sex, ethnicity (Hispanic or not), race (ie, white, Asian, native Hawaiian or Pacific Islander, black or African American, American Indian or Alaska Native, or unknown), and information about 2 economic indicators. Participants were asked how most of the student's dental care was paid for (ie, family or self-pay; Medicaid, medical card, Medicaid health maintenance organization, Healthy Start; other dental insurance; or don't know/don't remember) and whether the student was eligible for the FRPM program. The consent form asked 5 questions about oral health history and 1 about students' milk consumption.

### Data collection

In each county, we recruited health care professionals to be volunteer screeners through local programs of the Special Supplemental Nutrition Program for Women, Infants, and Children (WIC) and through local health departments; school nurses also volunteered. The volunteers' agencies allowed staff to participate during working hours.

ODH staff trained more than 300 health care professionals to be screeners. Training was conducted using a protocol for weighing and measuring based on *Guidelines for Measuring Heights and Weights and Calculation of Body Mass Index-for-Age in Ohio's Schools*, a document developed by ODH, School and Adolescent Health Program in 2003 (20). We conducted 5 regional training sessions and several ad hoc sessions. We trained all regional WIC directors on the protocol and asked them to train the professional health care staff at their local programs to serve as screeners. After completion of the training, volunteers signed a form indicating that they had completed the training and understood all of its components. Each screener signed a confidentiality statement, ensuring that the data collected would not be used for any purpose other than our study. We developed a training video to be used as a refresher course for all of the volunteers who had been trained.

After a screening was scheduled, we contacted volunteers in the school's county and designated a screener. We reviewed the screening protocol with the screener by telephone and faxed the screener an information sheet with screening time, directions to the school, and contact numbers, including emergency contact numbers for the oral health screener and for ODH staff who could conduct the screening in the event that the designated screener could not be present.

On the day of the screening, the BMI screener arrived 30 minutes before the screening to compile student consent forms and assemble the scale and stadiometer. The screener checked consent forms before each screening and rechecked the forms as students presented them before having their height and weight measured. Screeners recorded data in boxes located at the bottom of the consent form.

Students with consent were called in groups to the screening room. Screeners asked students to remove their shoes and heavy outer clothing and called students one at a time to check their teeth and measure their height and weight. Screeners never stated measurements aloud and shared them only confidentially with students if students requested their results. Screeners measured but did not record the height and weight of students in wheelchairs; who had amputations, casts, or nonremovable braces; or who were unable to stand or bear their own weight. Screeners gave stickers to students who participated in the BMI screening. The entire screening process lasted approximately 2 to 3 minutes per student.

### Measures

Screeners weighed students twice to the nearest 0.2 lb and recorded the average of the 2 weights. Screeners measured students' standing height once to the nearest 0.25 in. Students' measurements were obtained using Tanita electronic scales, model BWB-800 (Tanita Corporation of America, Inc, Arlington Heights, Illinois), and SECA portable stadiometers, model 214 (seca gmbh & co, Hamburg, Germany). The 13 sets of anthropometric equipment used in the survey were purchased and tested by ODH staff before the survey.

### Weighting

We incorporated adjustment for survey nonresponse into the sampling weights and incorporated poststratification adjustment for FRPM participation and race distribution into the final sampling weights for analysis.

First, we calculated anticipated sample weights and performed a nonresponse adjustment. We conducted a ratio adjustment to account for students who were eligible for inclusion but who were not screened. We segmented the full sample into weighting classes, which were determined by county groups. We computed adjustments and applied them to the anticipated weights of students who were screened so that these weights summed to the class weight total for third-grade students for both respondent and nonrespondent students. Adjustment factors ranged from 1.22 to 3.10. We set a cap of adjustment factors at 2.25, and 80% of the counties had an adjustment factor below this point. The nonresponse adjusted weight is the product of the adjustment factor and the anticipated sample weight.

Next, we calibrated the nonresponse adjusted weights to match the sum of nonresponse adjusted weights to the total number of third-grade students in the ODH data, on the basis of FRPM participation and race groups, to account for undercoverage of the population for each county. We made the adjustment to match FRPM and race enrollment totals, as reported by the ODE. For the cases in which FRPM status was missing or unknown by parents, we imputed FRPM participation. We used assignment to FRPM group (yes or no) only for poststratification classification of participants. We imputed missing race when information was not available from a chosen response category or a written-in "other" response. We grouped data for race into white, black, and other for weighting purposes. The poststratified adjusted weight is the product of the nonresponse adjusted weight and the poststratification adjustment factor*.*

Finally, in cases in which BMI was missing because of unknown sex, weight, height, or age, or in which the calculated BMI was out of the plausible range, we redistributed the observation's statistical weight within strata.

We made several assumptions when calculating the statistical weights. To generalize results of this study to the statewide population of third-grade students, we assumed that 1) the schools that refused to participate were not different from schools that did, 2) students who were eligible but not evaluated in each school (because of absenteeism or lack of consent) were not different with respect to oral health or BMI from students who were eligible and evaluated, and 3) during the 2004-2005 school year, 127,364 third-grade students were enrolled in Ohio public schools.

### Data management

We calculated percentiles for BMI-for-age and sex according to the 2000 CDC growth charts (21), using a CDC-provided SAS program (SAS Institute Inc, Cary, North Carolina), version 9.1 (www.cdc.gov/nccdphp/dnpa/growthcharts/resources/sas.htm).

We calculated age in months from the date of measurement and the reported date of birth. If birth date was not available but age in years was reported, we estimated age in months as the average age in months of all other students in the study who were also that age in years.

Outliers of the BMI-for-age percentile were flagged by the CDC-provided SAS program and are based on World Health Organization fixed-exclusion ranges (22). We defined outliers as a BMI-for-age percentile *z* score of less than −4 or greater than 5, and these were considered biologically implausible values.

This study was approved by the Ohio Department of Health Human Subjects Review Committee.

## Results

### Schools

During the 2004-2005 school year, 374 schools agreed to height and weight screening in Ohio. Of the 374 schools that were screened, 41 were considered to be replacement schools. Consent was not obtained in time for screening for 1 entire school district. As a result, we were unable to calculate county-level estimates for the county in which the school district was located.

### Students

Among participating schools (N = 374), third-grade enrollment as of the day of screening at each school was 26,590. Consent forms were returned by 17,557 (66.0%) third-grade students. Consent for BMI screening was granted for 15,209 (57.2%) students; of these students, 14,543 (54.7% of all enrolled third-grade students) were screened. Students who provided consent may not have been screened because of absence, refusal, or presence of a cast, missing limb, or inability to stand or bear their own weight. Valid information about sex or birth date was unavailable for 38 students, so BMI-for-age percentiles could not be determined. Fifty-one students (0.35% of students screened) had biologically implausible values (17 low and 34 high) for their BMI-for-age percentiles. These implausible values were dispersed among 33 of the 88 Ohio counties. Data to calculate BMI for 3 students were missing, and data on sex or date of birth of 38 students were invalid. Valid BMI-for-age percentile data were available for 14,451 students, resulting in an overall participation rate of 54.3%. The overall response rate of the oral health portion of the study was 52.8% in 2004-2005. The design effect associated with the statewide proportion of overweight or obese third-grade students was 4.9. The estimated interclass correlation coefficient was 0.10.

## Discussion

By adding BMI screening to Ohio's third-grade oral health survey and using trained volunteer screeners, ODH successfully implemented overweight and obesity surveillance using minimal resources. Combining surveys decreased the amount of money needed to design the survey and to collect and analyze data. The consolidation decreased intrusions on the school schedule and requests made to parents. The methods ensured appropriate communication with parents about the BMI status of their children. The success of the oral health survey was not compromised; its overall response rate was 52.8%, compared with a 57.5% overall response rate in a similar survey that included no BMI screening in 1998-1999 (17).

Our survey was of high quality for several reasons. First, we used a sound sampling methodology. Second, we collected directly measured anthropometric data rather than using self-reported or parentally reported heights and weights. Third, individuals trained in standard methods collected the data using standard equipment. Finally, we decreased response bias by applying statistical weights to all analyses.

The findings of this study are subject to several limitations. First, not all schools that were randomly chosen were willing to participate, and the health profiles of students in schools not willing to participate may differ from the profiles of students of participating schools. Second, we used a large number of screeners, but we did not formally certify them or perform any reliability checks on their activities; however, we did ask screeners to demonstrate their measuring techniques to an expert at each screening. Third, the sample selection was not initially designed for outcomes of overweight and obesity; therefore, precision may not be as high as it could have been if the survey had been designed for BMI. The BMI screening was added to the study following sampling, and the county-level estimates of BMI in this population needed for precise design were unknown. The findings of this survey will be used to ensure that future surveys have adequate precision for BMI. We now have a better estimate of nonresponse for this population and may consider sampling only some classes in each school to survey more schools and increase precision.

Finally, the low student response rate will introduce bias if the characteristics of nonparticipating students were not similar to those of participating students. The greatest reason for nonparticipation was nonreturn of consent forms; however, the reasons for nonreturn are unknown. Parents or guardians who did not complete the forms may have done so to actively deny consent. Alternately, desire to consent may have been unrelated to the return of forms. The response rate of this survey is similar to those of both New York State's BMI survey of elementary school-aged children, which had a 51% response rate (12), and Georgia's survey of third-grade students, which screened 49% of eligible students but had final data on only 38% of eligible students (23). Passive consent can produce substantially greater consent rates, ranging from 87% to 96% (13-16). With passive consent, however, no opportunity is provided for parentally reported information, such as race and behaviors.

Methods to improve the response rate will decrease the nonresponse bias in the population estimates when we are confident that appropriate adjustments for the remaining nonresponse rates are possible. The belief that students or families who refuse to participate are not like those who participate in terms of their BMI is reasonable, so we must adjust for nonresponse. Adjustments for nonresponse assume that the nonresponse rate can be ignored. This assumption may not be met, and these methods may fall short of accounting for all nonresponse bias incurred. Therefore, efforts must be improved in measuring auxiliary variables to characterize nonresponse. If nonresponse is substantial in certain classes or strata, the variance of estimates also will be increased. Although no guarantee exists that nonresponse bias will be low when we have a low nonresponse rate, we can minimize the risk of nonresponse bias by minimizing nonresponse and by being aware of auxiliary variables to measure or outside data sources to reference for adjustments (24). Reducing nonresponse and seeking as many auxiliary data as possible to use for nonresponse adjustment should be a high priority in future studies.

On the basis of the outcomes of the 2004-2005 survey, ODH will implement ongoing surveillance of childhood overweight and obesity through coordination with the oral health survey and will produce county-level estimates, supplemented annually with state-level estimates, approximately every 5 years. Future efforts should focus on methods to improve the student response rate.
